# Unexpected spontaneous expectoration of a grass bur: A case report

**DOI:** 10.1177/2050313X241274970

**Published:** 2024-08-22

**Authors:** Jacob A. Alaniz, Peyton Armstrong, Alexander Bosley, Vasilis Mavratsas, Randal Reinertson

**Affiliations:** 1 John Sealy School of Medicine, University of Texas Medical Branch, Galveston, TX, USA; 2 Department of Internal Medicine, University of Texas Medical Branch, Galveston, TX, USA

**Keywords:** Foreign body, aspiration, empyema, grass bur, pleuritic chest pain, chest tube

## Abstract

Adult foreign body aspiration is rare and represents only 15%–25% of all foreign body aspirations and 1 in 400 bronchoscopy procedures. Typically, adults present non-emergently and exhibit non-specific symptoms, which makes the diagnosis of foreign body aspiration especially difficult when a history of aspiration cannot be elicited. We present a 63-year-old male with a past medical history of chronic obstructive pulmonary disease hospitalized for left thoracic empyema caused by the aspiration of a grass bur. Our patient did not recall the aspiration event and the diagnosis was further obfuscated by a lack of radiographic evidence and other distracting disease processes. Thus, this case exemplifies the rationale for maintaining a suspicion of foreign body aspiration even for patients with little historical or radiographic evidence to support the presence of a foreign body. This is particularly salient for patients with a tumultuous hospital course or those who fail to respond to treatment.

## Introduction

Adult foreign body (FB) aspirations are rare and represent only 15%–25% of all FB aspirations and approximately 1 in 400 bronchoscopy procedures.^1,2^ Adults most commonly present non-emergently with symptoms consisting of cough (88%–96%), dyspnea (23%–35%), hemoptysis (4%–24%), fever (8%–12%), and chest pain (6%–15%) that result from distal impaction of the FB within the respiratory tree.^3,4^ The non-emergent and nonspecific nature of these symptoms can make diagnosing FB aspiration difficult, especially when the patient cannot provide a history of aspiration. We present a 63-year-old male with a past medical history of chronic obstructive pulmonary disease (COPD) hospitalized for left thoracic empyema caused by the aspiration of a grass bur.

## Case report

A 63-year-old man with previously diagnosed COPD (managed with budesonide/formoterol and tiotropium bromide) was hospitalized for a 2-week history of progressively worsening left-sided pleuritic chest pain, persistent cough, and yellow-blood-tinged sputum. The patient is a retired veteran living in a home with his spouse on Galveston Island. At baseline, he can complete his activities of daily living and tend to his household (including yard work and playing with his grandchildren) without difficulty. His symptoms acutely worsened at home, causing diaphoresis, nausea, and excruciating stabbing pain in the left chest and left upper abdomen, which brought him to the emergency department.

In the emergency room, a chest X-ray revealed a left lower lung zone airspace opacity suggestive of pneumonia. A computed tomography (CT) angiogram revealed severe centrilobular emphysema, left lower lobe consolidation with a small pleural effusion, and a 2.1 cm left lung hypodensity concerning pulmonary abscess. Reactive left hilar adenopathy was present. An electrocardiogram (EKG) performed in the emergency department was unremarkable.

On admission, he had decreased breath sounds, expiratory wheezing, and experienced bouts of chest pain that were exacerbated when belching. He was treated with analgesics, ampicillin/sulbactam, prednisone, ipratropium bromide/albuterol via nebulizer, and guaifenesin. Procalcitonin, sputum cultures, and urine antigens for Streptococcus and Legionella were collected. Pulmonology was consulted to evaluate the patient for thoracentesis.

Pulmonology placed a chest tube on the patient’s left side, which returned exudative fluid. He experienced mild improvement but continued to endorse chills, nausea, shortness of breath, and cough. Three hundred milliliters of yellow-colored fluid was collected over the next 3 days and the tube was removed due to low output, resolving white blood cell counts, and continued lack of fevers.

**Figure 1.**
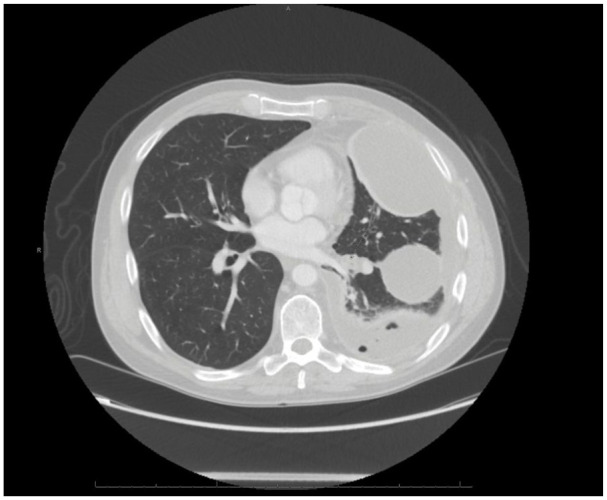
CT chest with contrast showing subtle filling of proximal left lower lobe bronchi without an obvious endobronchial lesion and left-sided empyema.

**Figure 2.**
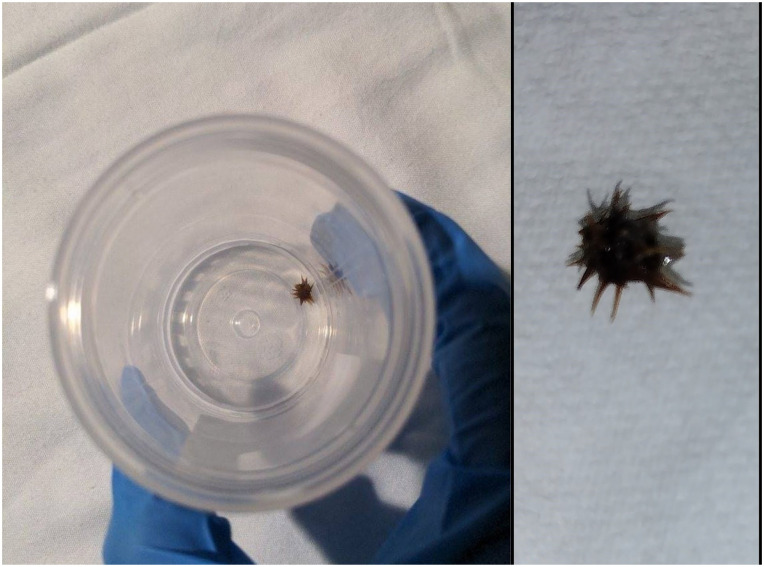
Sputum sample containing the grass bur expelled from the patient’s airway (left) and photo of bur taken by the patient (right).

The patient’s symptoms (including nausea, chills, and subjective fevers) quickly worsened, and it became too painful to expectorate sputum. A repeat chest X-ray and CT thorax (Figure 1) showed worsening of the pleural effusion and a new chest tube was placed the following day with flushes of Tissue Plasminogen Activator (TPA)/Deoxyribonuclease (DNase) every 12 hours. Over the next 2 days, 1650 mL of exudative fluid was collected and the patient’s condition mildly improved. Three days after chest tube placement, the patient complained of intense, worsening pain and expectorated a whole, intact grass bur after a prolonged bout of coughing (Figure 2). He reported near-immediate resolution of his symptoms and experienced no recurrence throughout the rest of his stay. A CT taken within 24 h showed that his pleural effusion had improved. The chest tube was discontinued the next day, and the patient was discharged shortly after on amoxicillin/clavulanate. CT thorax with contrast from March 2024 (approximately 5 months after his presentation to the emergency department) showed diffuse emphysematous changes and scattered parenchymal bands/scarring. No focal opacities were identified and no other pleural abnormalities were detected.

## Discussion

Grass burs are weeds that produce sharp, spiny burs in the summer and fall. The bur that was expelled from our patient’s airway appears similar to those from the Cenchrus genus (possibly *Cenchrus incertus* or *Cenchrus spinifex*). Although common in the southern United States, no case reports exist detailing the aspiration of a grass bur in an adult. After expelling the bur, our patient recalled a sharp object striking the back of his throat while using a string trimmer (“weed whacker”) 4–6 weeks prior. He remembered inspiring sharply and coughing vigorously, but his symptoms quickly resolved; he believed that the object had been expelled and initially provided no history of aspiration during his encounter. The patient’s symptoms were hypothesized to be sequelae of his chronic lung disease and empyema. However, the lack of notable improvement following antibiotic treatment and chest tube placement makes this less likely. On the other hand, the patient experienced near instantaneous improvement of his symptoms following the expectoration of the grass bur. He continued to do well at follow-up 1 week after discharge, making the bur the more likely culprit.

A post hoc analysis of the scans performed by radiology at our institution noted that the bur was difficult to detect since it is radiolucent and posited that it was likely located within one of the left lower lobe airways where endobronchial soft tissue material was seen in patient’s initial CTs. The consequence of this was post-obstructive pneumonia seen at his presentation, which cleared following the expectoration of the bur. Thus, the combination of nonspecific symptoms, non-emergent presentation, and lack of radiographic evidence or history supporting aspiration created difficulties in establishing the diagnosis.

The available literature seems to support that spontaneous expectoration of foreign bodies is a rare event in both the pediatric and adult populations. The general factors that seem to contribute to the likelihood of expulsion from the airway include the following: the length of time the object has been within the airway, the location of the aspirated object, and the physical characteristics of the object itself. In adults, aspirated objects are more likely to travel more distally into the airways when compared to pediatric patients. Thus, foreign bodies must travel a greater distance to be expelled, which offers greater opportunities to become stuck. Objects that remain in the airway for extended periods of time are subject to the formation of inflammation, granulation tissue, and scar tissue, which can further impede their movement. Furthermore, objects made of organic matter or those with sharp/jagged edges may be uniquely difficult to expel since they cause greater mucosal inflammation and can puncture the mucosa of the airway.^5–7^ Other considerations include the ability of the patient to produce a forceful cough and whether their cough reflex is stimulated by the FB (or present altogether).

A review of the available literature also demonstrated that delayed diagnosis of adult FB aspiration is common and can be attributed to the same pitfalls affecting this case. As many as 46%–58% of adults suffering from FB aspiration are not diagnosed until at least 1 month after aspiration.^4^ Furthermore, bronchoscopy data from 1974 to 2014 revealed that only 25% of the patients greater than 12 years old presented within the first 7 days following aspiration.^8^ Similarly, Ng et al. reported that, of the 103 patients in their study who underwent bronchoscopy to remove airway FB, only 56% of patients presented to the hospital for persistent respiratory symptoms immediately following the aspiration event.^9^ This is further complicated by the potential for patients to fail to supply a history of aspiration during their first encounter with a physician. In a retrospective analysis performed by Lin et al., only 5 of 17 geriatric patients (>65 years old) could supply a history of aspiration. Similarly, only 13 of 26 non-geriatric patients (18–64 years old) could supply a history of aspiration.^3^ This case represents a unique combination of these phenomena.

## Conclusion

In summary, we describe a case of accidental inhalation of a grass bur in an adult male with chronic lung disease. Due to difficulties obtaining a history of aspiration, lack of radiographic evidence, and other distracting disease processes, FB aspiration was not suspected to be the source of the patient’s symptoms. Although spontaneous expectoration is rare, the combination of expectorants, bronchodilators, analgesic therapy, steroid therapy, and pulmonary toilet likely brought the grass bur into the proximal airway where a vigorous cough could expel it from the respiratory tract. Our case exemplifies the rationale for maintaining a suspicion of FB aspiration even for patients with little historical or radiographic evidence to support the presence of an FB. This is particularly salient for patients with a tumultuous hospital course or those who fail to respond to treatment.
